# Hydrocortisone enhances the barrier properties of HBMEC/ciβ, a brain microvascular endothelial cell line, through mesenchymal-to-endothelial transition-like effects

**DOI:** 10.1186/s12987-015-0003-0

**Published:** 2015-03-05

**Authors:** Tomomi Furihata, Shinya Kawamatsu, Ryo Ito, Kosuke Saito, Shota Suzuki, Satoshi Kishida, Yoshiro Saito, Atsuko Kamiichi, Kan Chiba

**Affiliations:** Laboratory of Pharmacology and Toxicology, Graduate School of Pharmaceutical Sciences, Chiba University, 1-8-1 Inohana, Chuo-ku, Chiba-shi, Chiba 260-8675 Japan; Division of Medical Safety Science, National Institute of Health Sciences, 1-18-1 Kamiyoga, Setagaya Tokyo, Japan

**Keywords:** Blood–brain barrier, Brain microvascular endothelial cells, *In vitro* BBB model, Hydrocortisone, Mesenchymal-to-endothelial transition, Adherens junction, Plasmalogen

## Abstract

**Background:**

Because *in vitro* blood–brain barrier (BBB) models are important tools for studying brain diseases and drug development, we recently established a new line of conditionally immortalized human brain microvascular endothelial cells (HBMEC/ciβ) for use in such models. Since one of the most important functional features of the BBB is its strong intercellular adhesion, in this study, we aimed at improving HBMEC/ciβ barrier properties by means of culture media modifications, thus enhancing their use for future BBB studies. In addition, we simultaneously attempted to obtain insights on related mechanistic properties.

**Methods:**

Several types of culture media were prepared in an effort to identify the medium most suitable for culturing HBMEC/ciβ. The barrier properties of HBMEC/ciβ were examined by determining Na^+^-fluorescein permeability and transendothelial electric resistance (TEER). Endothelial marker mRNA expression levels were determined by quantitative real-time polymerase chain reaction. Adherens junction (AJ) formation was examined by immunocytochemistry. Cell migration ability was analyzed by scratch assay. Furthermore, cellular lipid composition was examined by liquid chromatography-time-of-flight mass spectrometry.

**Results:**

Our initial screening tests showed that addition of hydrocortisone (HC) to the basal medium significantly reduced the Na^+^-fluorescein permeability and increased the TEER of HBMEC/ciβ monolayers. It was also found that, while AJ proteins were diffused in the cytoplasm of HBMEC/ciβ cultured without HC, those expressed in cells cultured with HC were primarily localized at the cell border. Furthermore, this facilitation of AJ formation by HC was in concert with increased endothelial marker mRNA levels and increased ether-type phosphatidylethanolamine levels, while cell migration was retarded in the presence of HC.

**Conclusions:**

Our results show that HC supplementation to the basal medium significantly enhances the barrier properties of HBMEC/ciβ. This was associated with a marked phenotypic alteration in HBMEC/ciβ through orchestration of various signaling pathways. Taken together, it appears that overall effects of HC on HBMEC/ciβ could be summarized as facilitating endothelial differentiation characteristics while concurrently retarding mesenchymal characteristics.

**Electronic supplementary material:**

The online version of this article (doi:10.1186/s12987-015-0003-0) contains supplementary material, which is available to authorized users.

## Background

The blood–brain barrier (BBB), which is formed primarily by brain microvascular endothelial cells (BMECs), is an interface between the central nervous system (CNS) and the systemic circulation [1]. Several cell types located adjacent to BMECs (including astrocytes and pericytes) are also known to contribute to BBB function. One of the most important features of the BBB is its extremely strong intercellular adhesion, which is established by adherens junctions (AJs) and tight junctions (TJs) between the endothelial cells [1]. This forceful adhesion seals the paracellular route and prevents entry of a variety of substances, both small and large, into brain from blood, while simultaneously creating a foundation that allows BMEC transporters to take up or expel molecules indispensable for, or harmful to, the physiological functions of the brain.

Based on this functional importance in maintaining brain homeostasis, it has become increasingly evident that impairment of BBB function is associated with various CNS diseases, such as multiple sclerosis, amyotrophic lateral sclerosis and Alzheimer’s disease, even though it remains currently inconclusive whether BBB impairment is a cause or consequence of those diseases [2,3]. On the other hand, because the BBB appears to prevent passage of more than 98% of all small therapeutic molecules [4], the barrier is considered a primary obstacle that prevents drugs from exercising their pharmacological actions in brain. This is a critical reason why the development of CNS drugs is difficult and time consuming. Collectively, the BBB is a pivotal research target for various brain diseases and CNS drug development studies.

*In vitro* BBB models are among the most important tools used in BBB studies [5,6]. While it is probably inevitable that *in vitro* BBB models will never reflect the full range of *in vivo* BBB functionalities due to their differing environments, such models offer multiple experimental benefits in terms of simplicity, scalability, and versatility. In *in vitro* BBB models, BMECs are cultured on a porous membrane in a transwell culture system, thereby resulting in a cell layer that creates separate blood-side and brain-side compartments. To date, human and animal primary BMECs, as well as immortalized human and animal BMECs, have been extensively utilized in such models [6]. The primary cells show excellent functionality, but they suffer from several experimental limitations, such as scarcity, low cell proliferation potential and sample to sample variations. On the other hand, even though the functional levels of immortalized cells are not regarded as being as high as that of the freshly isolated primary cells, they show infinite proliferation ability and stable phenotypes, which makes them useful to researchers conducting a variety of experiments. Therefore, it would be ideal if, through the refinement and elaboration of their culture methods, the BBB functions of immortalized cells could be improved to levels that are comparable to primary cells.

Recently, we reported establishment of a new line of human immortalized BMECs, HBMEC/ciβ [7]. In addition to excellent proliferative ability, HBMEC/ciβ express a series of endothelial marker genes (e.g., von Willebrand factor (vWF) and vascular endothelial-cadherin (VE-cadherin)) along with BBB-related genes (e.g., claudin-5, glucose transporter 1, P-glycoprotein, and transferrin receptor), and limited sucrose and Na^+^-fluorescein (Na-F) penetration across cell monolayers. Based on these characteristics, it is logical to conjecture that HBMEC/ciβ have significant potential for providing the foundation of uniquely effective *in vitro* BBB models. However, to achieve this goal, further improvements to the intercellular junctional property of the cells are absolutely necessary.

Since it is well known that the barrier function of *in vitro* BBB models can be reinforced or degraded by various biological and chemical factors [6], it is also likely that the culture media composition plays a crucial role in determining the strength of the HBMEC/ciβ barrier function. In this regard, it was noted that the culture medium we used initially was designed for pan-primary cells, which caused us to consider the possibility that media optimization for HBMEC/ciβ culture might enhance barrier properties. Accordingly, the primary purpose of the present study was to clarify the effects of culture media modifications on HBMEC/ciβ barrier functions. In addition, we provide results that show mechanistic insights into the effects of those modifications.

## Methods

### Culture medium

CSC medium (Complete Medium Kit containing 10% fetal bovine serum, 4Z0-500-R, Cell Systems, Kirkland, WA, USA) or EBM2 medium (Lonza, Walkersville, MD, USA) was used as a basal medium. CultureBoost-R (cbR, 2% v/v, Cell Systems) and SingleQuots (SQs, Lonza) were used as culture supplements. Although the components of cbR are not published, according to the manufacturer’s information it contains several growth factors. SQs consist of a series of vials, each of which contains 2% (v/v) fetal bovine serum, 0.1% (v/v) vascular endothelial growth factor, 0.1% (v/v) long R3 insulin-like growth factor, 0.1% (v/v) recombinant human epidermal growth factor, 0.4% (v/v) recombinant human basic fibroblast growth factor, 0.1% (v/v) hydrocortisone (HC), 0.1% (v/v) heparin or 0.1% (v/v) ascorbate. The actual HC concentration is 180 nM based on our determination using enzyme-linked immunosorbent assay. Additionally, all culture media were supplemented with blastcidin S (4 μg/mL) and penicillin-streptomycin. Medium information is also provided in Additional file 1: Figure S1.

### Cell culture

HBMEC/ciβ were routinely grown on type-I collagen-coated dishes in CSC-cbR at 33°C with 5% CO_2_/95% air. They were seeded (day 0) at 1.0 × 10^5^ or 4.0 × 10^5^ cells/mL onto a dish or a membrane filter of an insert culture system (polyethylene terephthalate, 0.4 μm high-density pores, and 0.3 cm^2^, BD Falcon, Franklin Lakes, NJ, USA), respectively. At day three, the medium was changed to either the same or a differently supplemented medium, depending on the experiments (for example, the medium was changed from CSC-cbR to CSC-HC at day three). Then, the cells were continuously cultured for 12 days, during which a medium change was conducted every other day. All functional or gene expression analyses were performed on day 12. The culture schedule is also provided in Additional file 1: Figure S1.

### Permeability assay

The apparent permeability (P_app_, cm/min) and the permeability coefficients (P_e_, cm/min) of Na-F (Sigma, St. Louis, MO, USA) and [^14^C] sucrose (GE Healthcare, Giles, UK) were determined essentially as described previously [7]. Briefly, the medium was replaced with serum-free CSC medium 30 min before the assay (4Z3-500-R, Cell Systems). The assay was initiated by adding [^14^C] sucrose (0.1 μCi/mL) or Na-F (500 ng/mL) to the insert at 37°C. The incubation time was 40 min. The P_e_ values were calculated using the permeability-surface area product (PS, μL/min) described in our previous report [7], and the P_app_ values were calculated using the following equation:

$$ {P}_{app}\left( cm/ min\right)=\frac{V_{baso}\ \left(c{m}^3\right)}{\mathrm{A}\ \left(c{m}^2\right)\times {\left[{C}_0\right]}_{api}\ \left( ng/\mu L\right)}\times \frac{\varDelta {\left[C\right]}_{baso}\ \left( ng/\mu L\right)}{\varDelta T\ (min)} $$

where *V*_*baso*_ is medium volume at the basolateral side, *A* is membrane surface area (0.3 cm^2^), *[C*_*0*_*]*_*api*_ is Na-F concentration at the apical side at T = 0, *[C*_*0*_*]*_*baso*_ is Na-F concentration at the basolateral side at T = 40, and *ΔT* is time of experiment.

### Determination of transendothelial electric resistance (TEER)

TEER was examined using the Millicell ERS-2 (Millipore, Billerica, MA, USA) before performing permeability analysis. After subtracting the ohms of a blank insert membrane from the ohms of cell monolayer, the value was multiplied by 0.33 cm^2^ (Ω × cm^2^).

### Total RNA isolation, cDNA synthesis, and quantitative real-time polymerase chain reaction (qPCR)

Total RNA extraction and cDNA synthesis of the HBMEC/ciβ (cultured as described above) were conducted using methods described previously [7]. qPCR was performed using the previously-described SYBR green-based method [7] to determine the following mRNA expression levels: glucocorticoid-induced leucine zipper (GILZ), nuclear factor-Kappa B inhibitor alpha (NFκBIA), annexin A1 (ANXA1), matrix metalloproteinase 1 (MMP-1), MMP-2, MMP-16, vWF, Duffy antigen/receptor for chemokines (DARC), angiopoietin 2 (ANGPT2), early growth response 1 (EGR-1), histone deacetylase 7 (HDAC7), inhibitor of differentiation or DNA binding-1 (Id-1), Ras-proximate-1 or Ras-related protein 1 (RAP1), exchange protein directly activated by cAMP (EPAC), VE-cadherin, claudin-5, occludin, glyceronephosphate O-acyltransferase (GNPAT), alkylglyceronephosphate synthase (AGPS), fatty acyl-CoA reductase 1 (FAR1), and glyceraldehyde 3-phosphate dehydrogenase (GAPDH). The primers used for qPCR are described in (see Additional file 2: Table S1), and the amplification efficiency of each PCR was confirmed to be close to one. Data was calculated using the delta-delta-CT method, where GAPDH was used as a control.

### Western blotting analysis

HBMEC/ciβ cells were cultured on dishes as described above. Homogenates were prepared using methods described previously [7]. Proteins were separated by sodium dodecyl sulfate-polyacrylamide gel electrophoresis, and then transferred onto a polyvinyldene difluoride membrane. The membrane was blocked with 5% skim milk. The primary antibodies used were rabbit anti-glucocorticoid receptor (GR) polyclonal IgG (1,000-fold dilution, sc-1002, Santa Cruz Biotechnology, Santa Cruz, CA, USA), rabbit anti-VE-cadherin polyclonal IgG (1,000-fold dilution, sc-28644, Santa Cruz Biotechnology), rabbit anti-claudin-5 polyclonal IgG (1,000-fold dilution, ab53765, Abcam, Cambridge, UK), or rabbit anti-occludin polyclonal IgG (1,000-fold dilution, 71–1500, Zymed Laboratories, San Francisco, CA, USA). The secondary antibody used was goat anti-rabbit IgG–peroxidase antibody (10,000-fold dilution, A9196, Sigma).

### Fluorescence detection of adherens junction-related proteins

The insert membrane filter on which HBMEC/ciβ were cultured was taken out, followed by incubation with BD Cytofix/Cytoperm Fixation and Permeabilization Solution (BD Biosciences) for 40 min at 4°C (fixation). The membrane was then incubated with BD Perm/Wash Buffer (BD Biosciences) for 15 min at room temperature (permeation), and blocked with BLOCKACE (DS Pharma Biomedical, Osaka, Japan) for 30 min. For immunocytochemistry, the primary antibodies used were anti-VE-cadherin rabbit polyclonal IgG (100-fold dilution, sc-28644, Santa Cruz Biotechnology), anti-β-catenin rabbit monoclonal IgG (100-fold dilution, #8480, Cell Signaling Technology, Danvers, MA, USA), and anti-zonula occludens-1 (ZO-1) rabbit polyclonal IgG (100-fold dilution, #5406S, Cell Signaling Technology). The secondary antibodies used were Rhodamine (TRITC)-AffiniPure F(ab’)2 Fragment goat anti-rabbit IgG (100-fold dilution, Jackson Immuno Research Laboratories, West Grove, PA, USA) or Alexa Fluor 488 donkey anti-rabbit IgG (100-fold dilution, Life Technologies). The above antibodies were diluted to the indicated concentrations with CanGetSignal immunostain solution A (TOYOBO, Osaka, Japan). It was confirmed that the secondary antibodies did not bind to cellular proteins in a non-specific manner. For F-actin detection, the membrane was incubated with Acti-stain 488 Fluorescent Phalloidin (150-fold dilution, Cytoskeleton, Denver, CO, USA) for 30 min at room temperature. Fluorescence was detected using the OLYMPUS LSM (Olympus, Tokyo, Japan). The above immunocytochemical analyses were also performed in the presence of GGTI298 (5 μM, Sigma), a RAP inhibitor. The inhibitor or its vehicle (DMSO) was added during every medium change (Days 3, 5, 7, 9 and 11).

### Scratch assay

HBMEC/ciβ cells were cultured on dishes as described above. A scratch on the cell monolayer was created using a tip, immediately after which the medium was changed to wash away the floating cells (time = 0). Cell migration status was observed at time = 0, 6, and 12 hrs. The width of the scratch was calculated using Motic Image Plus 2.2S (Shimadzu, Tokyo, Japan).

### Lipidomics analysis

HBMEC/ciβ cells were cultured on dishes with CSC-free or CSC-HC. The cells were also cultured with CSC-HC containing RU486. After twelve days of culturing (at the confluent status), cells were washed twice with phosphate buffered saline (PBS), and collected. Lipids, corresponding to half of one dish, were extracted from the cells with 200 μL of methanol with internal standards (2 μM of 12:0/12:0 phosphatidylcholine (PC) [Avanti Polar Lipids, Alabaster, AL, USA] for PC, 2 μM of 12:0/12:0 phosphatidylethanolamine (PE) [Avanti Polar Lipids] for PE and 0.5 μM of d18:1/17:0 sphingomyelin (SM) [Avanti Polar Lipids] for SM). Next, filtering was performed for the following non-targeted measurement of PC, ether-type PC (ePC), PE, ether-type PE (ePE), and SM by liquid chromatography-time-of-flight mass spectrometry (LC-TOFMS; ACQUITY UPLC System [Waters, Milford]-LCT Premier XE [Waters, Milford]), as described previously [8]. The relative standard deviation of the internal standards (PC, PE and SM), which monitor experimental quality throughout extraction and measurement, were 5.6%, 7.9% and 9.7%, respectively.

Raw data obtained by LC-TOFMS were processed using 2DICAL software (Mitsui Knowledge Industry, Tokyo, Japan), which allows detection and alignment of the ion peaks of each ionized biomolecule obtained at the specific m/z and column retention time (RT). The main 2DICAL parameter was set as described previously with a few modifications [8]. The RT range was set from 2.0 to 38.0 min in the negative ion mode in order to extract the ion peaks. Ion peaks with the top 300 signal intensities were set as the cut-off and were used in the following data analyses. Extracted ion peaks were subjected to identification of lipid molecules by comparison of ion features, including RT, m/z, preferred adducts, and in-source fragments, of the experimental samples with those of our reference library of lipid molecule entries, as described previously [8]. Processing of extracted ion peaks yielded 104 lipid molecules, including 40 PC, 7 ePC, 26 PE, 21 ePE and 10 SM (see Additional file 3: Table S2). To determine the amount of each lipid molecule, the intensities of each extracted ion peak were normalized to those of the internal standards.

### Statistical analysis

One-way analysis of variance was first performed to determine whether there was a significant difference among values, after which a Student’s *t*-test was performed to determine statistical significance of difference between values. A statistical software package (Statcell, OMS, Saitama, Japan) was used for these analyses.

## Results

### Culture media composition differentially affected junctional properties of HBMEC/ciβ

Several media that have been optimized for endothelial cell culture are currently commercially available. Taking advantage of the successful use of these media in endothelial cell culture, we conducted preliminary screenings to identify the medium most suitable for HBMEC/ciβ culture. Although the results are not shown here, it was determined that, among the media, the EBM basal 2 medium supplemented with SingleQuots (EBM2-SQs) provided differential effects on the morphology and gene expression profile of HBMEC/ciβ when compared to those cultured with our initially-used medium, which was a CSC-complete recombinant serum-containing medium supplemented with cbR (CSC-cbR). (Please note that medium is called “basal medium-supplement” throughout the manuscript.)

**Figure 1:**
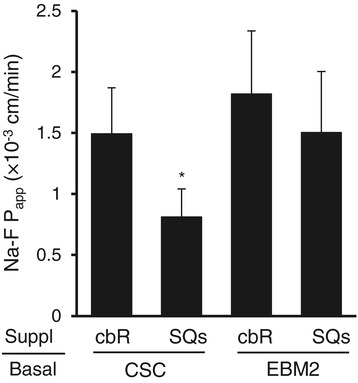
**Effects of different basal medium and supplement combinations on Na-F permeability in HBMEC/ciβ-based blood–brain barrier model.** Four media types were prepared, where either CSC or EBM2 basal medium (indicated by “Basal”) was supplemented by either cbR or SQs (indicated by “Suppl”). Three days after cell seeding, the initial medium was changed to one of the above-mentioned media. The cells were cultured continuously for 12 days, after which permeability assay was performed. Na-F permeability (P_app_, × 10^−3^ cm/min) was expressed as mean ± S.D. of three independent experiments. The asterisk indicates *p* < 0.05 compared to the value of CSC-cbR.

We then proceeded to examine the effects of different combinations of basal media and culture supplements (CSC-cbR, CSC-SQs, EBM2-cbR and EBM2-SQs) on Na-F permeability to determine whether either EBM-2 and SQs or both would affect HBMEC/ciβ barrier properties (Figure 1). The results showed that neither EBM2-cbR nor EBM2-SQs improved the junctional properties of HBMEC/ciβ over the levels obtained using CSC-cbR. However, CSC-SQs significantly decreased the Na-F permeability of HBMEC/ciβ. Therefore, it appeared that SQs was effective in strengthening the barrier property of HBMEC/ciβ, while the CSC basal medium was also essential.

### Hydrocortisone was identified as a component of SQs responsible for improving HBMEC/ciβ barrier properties

**Figure 2:**
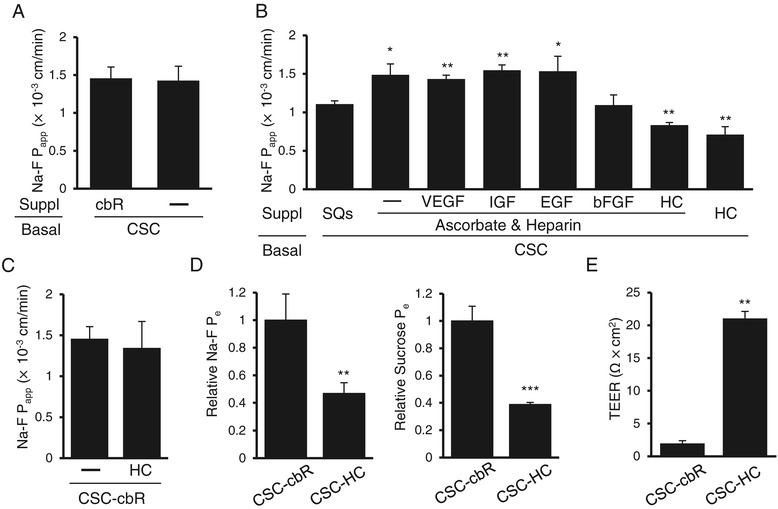
**Identification and characterization of barrier strengthening effects of hydrocortisone on HBMEC/ciβ-based blood–brain barrier model. (A)** The effect of cbR withdrawal from the culture medium on HBMEC/ciβ barrier properties was examined. The “-” symbol indicates the absence of cbR. **(B)** SQs components were screened for identification of the factors responsible for barrier property improvement. Here, the “-” symbol indicates the absence of growth factors. **(C)** The effect of cbR on the HC-mediated decrease in Na-F permeability of HBMEC/ciβ was examined. Here, the “-” symbol indicates the absence of HC. **(D)** The effect of HC on barrier function of HBMEC/ciβ was confirmed by determining the relative P_e_ values of Na-F (left) and sucrose (right) permeability. The value obtained from the cells cultured with CSC-HC was calculated relative to the value obtained from CSC-cbR cells (set as basal level =1) in each assay. **(E)** TEER was examined and compared between HBMEC/ciβ cultured with CSC-cbR and CSC-HC. The values of Na-F permeability (P_app_ or P_e_) (×10^−3^ cm/min) and TEER (Ω × cm^2^) were expressed as mean ± S.D. of three independent experiments. The single, double, and triple asterisks indicate *p* < 0.05, *p* < 0.01, and *p* < 0.005, respectively, compared to the value of CSC-SQs in **(B)** or CSC-cbR in **(D)** and **(E)**.

It was expected that improvements in HBMEC/ciβ barrier properties by CSC-SQs culture conditions would be due to either the withdrawal of cbR from or the addition of SQs to the CSC basal medium. To clarify which was responsible, we first compared barrier properties of cells cultured with CSC-cbR to those cultured with CSC basal medium alone (CSC-free). The results showed the Na-F permeability levels were comparable to each other (Figure 2A), thus suggesting that SQs were likely to play the primary improvement role in the HBMEC/ciβ barrier property.

SQs consists of several culture support agents. To identify the primary component(s) of SQs that was responsible for favorable effects on HBMEC/ciβ barrier properties, individual factors were examined separately (Figure 2B). Among those factors, HC alone (180 nM) was found to be effective in strengthening HBMEC/ciβ barrier function. The Na-F P_app_ value of the cells cultured with CSC-HC was 0.71 ± 0.10 (×10^−3^ cm/min), which was significantly lower than that of CSC-cbR (1.46 ± 0.15 [×10^−3^ cm/min]). Next, the cooperative actions of cbR or other SQ factor(s) with HC on barrier tightening were tested. However, no combination showed effects that were superior to that of HC alone (Figure 2C and see Additional file 4: Figure S2).

To further verify the barrier-strengthening effect of HC, Na-F and sucrose P_e_ values, along with TEER, were determined. Results showed that Na-F and sucrose P_e_ values of cells cultured with CSC-HC were less than 40% of those cultured with CSC-cbR (Figure 2D), and that TEER values were more than 10-fold higher in cells cultured with CSC-HC compared with that of CSC-cbR (Figure 2E). Taken together, the results showed that HC was a primary component in the SQs that could enhance the HBMEC/ciβ barrier function.

### Glucocorticoid receptor was involved in hydrocortisone-mediated functional improvements in HBMEC/ciβ barrier properties

**Figure 3:**
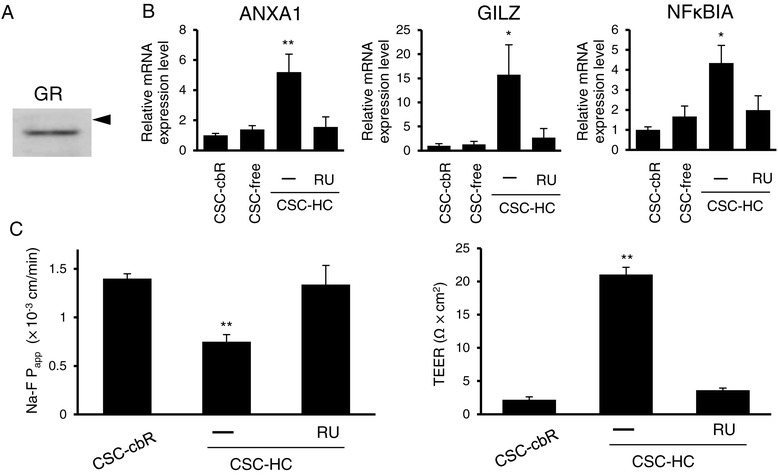
**Involvement of glucocorticoid receptor in the effects of hydrocortisone on HBMEC/ciβ barrier function. (A)** GR protein expression was examined by Western blot analysis. The arrowhead indicates 100 kDa. **(B)** mRNA expression level of representative GR-target genes (ANXA1, GILZ, and NFκBIA) in cells cultured with CSC-cbR, CSC-free, or CSC-HC were examined by qPCR. Effect of a GR antagonist, RU486 (RU), on HC-mediated induction of mRNA expression was also investigated. mRNA expression levels were calculated relative to the value obtained from CSC-cbR cells (set as the basal level =1). **(C)** Effects of RU486 on HC-mediated barrier reinforcement was examined by determining Na-F permeability (left) and TEER (right). Each value in the above experiments is expressed as mean ± S.D. obtained from three independent experiments. In **(B)** and **(C)**, the “-” symbol indicates DMSO (0.1%) was added to the medium as a control. The single and double asterisks indicate *p* < 0.05 and *p* < 0.01, respectively, compared to the value of CSC-free in **(B)** or CSC-cbR in **(C)**.

It is well known that HC is a ligand of the nuclear receptor, GR, and that binding of HC to GR stimulates its translocation to the nucleus, where GR modulates gene expression [9]. This is considered to be the primary pathway via which HC elicits pleiotropic effects on cellular function. To clarify the involvement of GR in HC-mediated improvements in HBMEC/ciβ barrier properties, the functional expression of GR was first characterized. Western blotting analysis showed that GR protein expression was clearly detected in HBMEC/ciβ (Figure 3A). In addition, qPCR showed that mRNA levels of known GR-target genes, GILZ [10], NFκBIA [11,12], and ANXA1 [13,14], were significantly increased in HC-containing medium (Figure 3B). Furthermore, increased mRNA expression of these genes was prevented by addition of RU486 (250 nM), a GR-antagonist. Given this clarification of functional GR expression in HBMEC/ciβ, the effects of RU486 on the Na-F permeability and TEER of these cells were examined. RU486 treatment nullified the barrier-tightening effects of HC (Figure 3C). These results indicate that GR plays a crucial role in HC-mediated improvements in HBMEC/ciβ barrier properties.

### Adherens junction formation was facilitated by hydrocortisone

**Figure 4:**
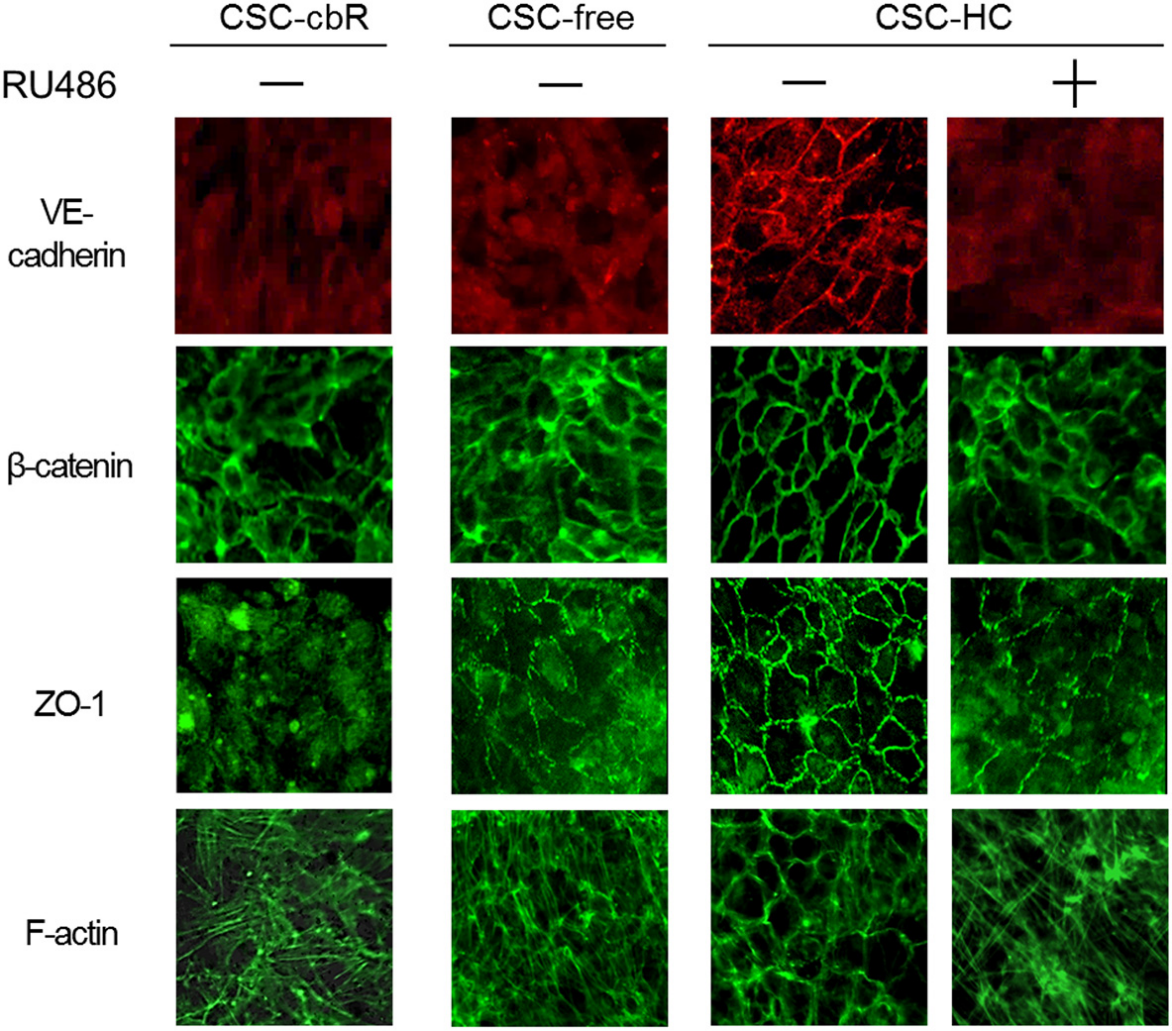
**Immunocytochemical analysis of adherens junction proteins and the effect of hydrocortisone.** Immunocytochemistry was performed to identify the cellular localization of VE-cadherin, β-catenin, and ZO-1 in cells cultured with CSC-cbR, CSC-free, or CSC-HC. Phalloidin staining was performed to analyze F-actin distribution. The effect of RU486 on the distribution of the junction-related proteins was also investigated in cells cultured with CSC-HC. The experiments were repeated six times, and representative results are shown: “+” or “-” symbols indicate the addition of RU486 or DMSO (0.1%) to the medium, respectively.

To further clarify the mechanisms underlying the barrier-strengthening effect of HC, the status of intercellular junction formation in HBMEC/ciβ cultured with each medium was examined by immunocytochemistry for VE-cadherin and β-catenin, which are representative components of AJ [15], and ZO-1, which is an important molecule for both AJ and TJ [16,17] (Figure 4). The results showed that VE-cadherin, β-catenin and ZO-1 were localized at the cell-to-cell border membrane in cells cultured with CSC-HC, while they primarily diffused throughout the cytoplasm in cells cultured with CSC-cbR and CSC-free. The plasma membrane localization in cells cultured with CSC-HC was significantly impaired by RU486 treatment.

In addition, the intracellular organization of F-actin was determined using fluorescently labeled phalloidin, because it has been known that F-actin undergoes structural remodeling to localize to the peri-plasma membrane region, where it can stabilize AJ [18]. The results of phalloidin-staining showed that F-actin appeared concentrated in the vicinity of the plasma membrane in cells cultured with CSC-HC, but not in those cultured with other media. Similar to the junctional proteins, this circumferential organization was severely disrupted by RU486 treatment. Because it was also possible that HC enhanced VE-cadherin expression in HBMEC/ciβ, qPCR and Western blotting analyses were performed. However, no increase in VE-cadherin mRNA or protein was observed with HC (see Additional file 5: Figure S3).

Tight junctions play a crucial role in BBB function [1,19], therefore, qPCR and Western blot analyses were performed to examine the key TJ proteins, claudin-5 and occludin, [19,20]. However, no changes in claudin-5 or occludin protein expression levels were observed in the presence of HC, even though occludin mRNA levels were increased (see Additional file 6: Figure S4). Next, immunocytochemical analyses of claudin-5 and occludin were performed. However, those analyses showed that they were not clearly identified at the cell border, thus indicating tight junction immaturity (data not shown). These results suggested that facilitation of AJ formation in HBMEC/ciβ was likely related to barrier function improvements by HC.

### The EPAC-RAP1 pathway is involved in hydrocortisone-mediated facilitation of adherens junction formation in HBMEC/ciβ

**Figure 5:**
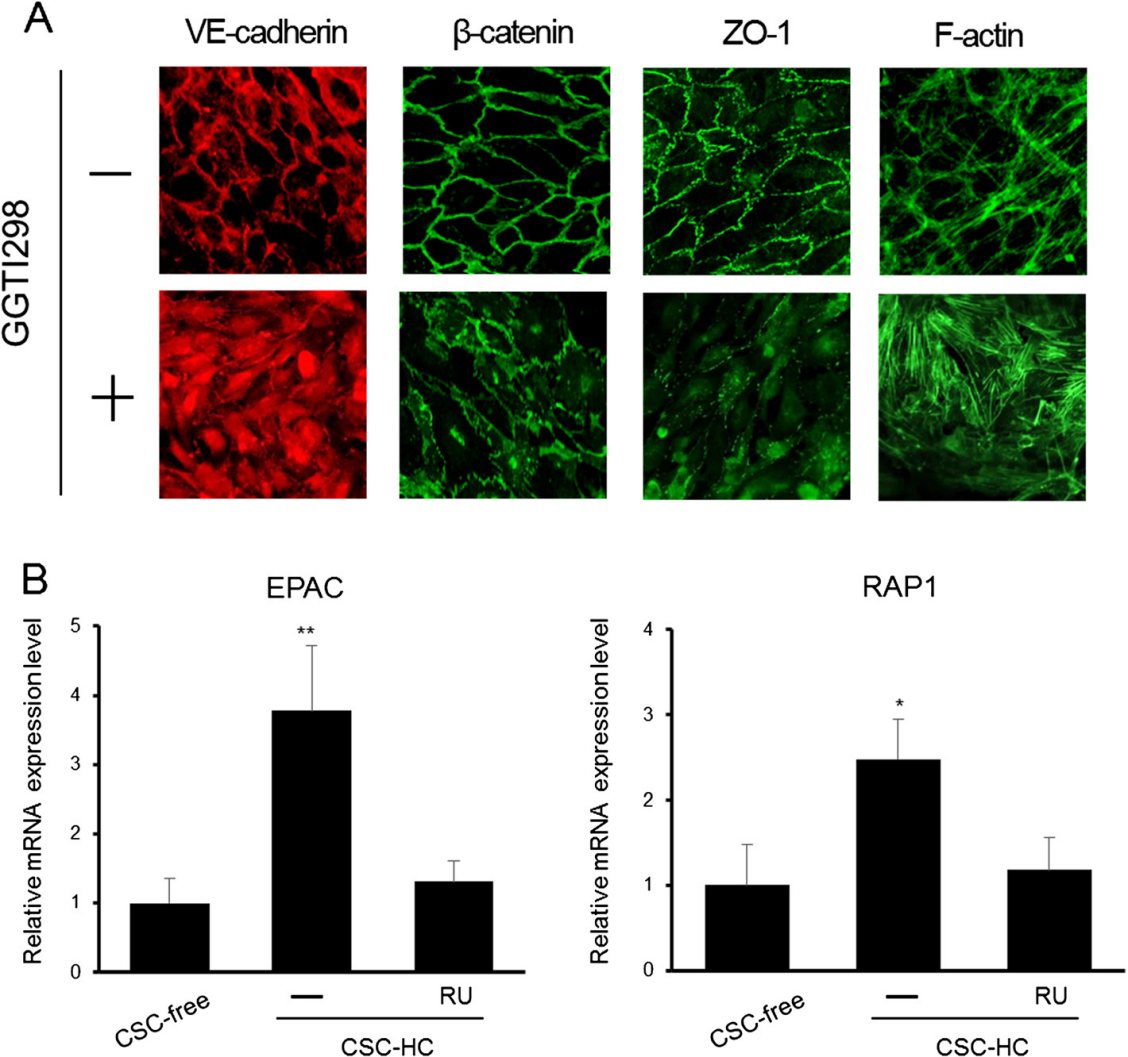
**Impairment of hydrocortisone-mediated facilitation of adherens junction formation by inhibiting RAP1 activity. (A)** Immunocytochemistry was performed to examine the cellular localization of VE-cadherin, β-catenin, and ZO-1 in cells cultured with CSC-HC in the presence or absence of a RAP1 inhibitor, GGTI298 (5 μM). Phalloidin staining was performed to analyze F-actin distribution. The experiments were repeated three times and representative results are shown. The “+” or “-” symbols indicate the addition of GGTI298 or DMSO (0.1%) to the medium, respectively. **(B)** EPAC and RAP1 mRNA expression levels in cells cultured with CSC-free or CSC-HC were examined by qPCR. Effect of RU486 on HC-mediated induction of mRNA expression was also investigated. mRNA expression levels were calculated relative to the value obtained from CSC-free cells (set as the basal level =1). Each value is expressed as mean ± S.D. obtained from three independent experiments. The single and double asterisks indicate represent *p* < 0.05 and *p* < 0.01 respectively, relative to CSC-free.

The HC-mediated facilitation of AJ formation described above is reminiscent of findings that show the EPAC-RAP1 pathway plays a pivotal role in AJ formation by recruiting AJ proteins to the plasma membrane [21]. Thus, to explore the relationship between these two events, the effect of GGTI298 (a RAP1 inhibitor) on the actions of HC in HBMEC/ciβ was examined. Results showed that in GGTI298-treated cells, VE-cadherin, β-catenin, and ZO-1 were dispersed intracellularly, which clearly differs from their cellular localization profiles in cells with CSC-HC (Figure 5A). Consistently, the lowered Na-F permeability with HC was completely reversed in the presence of GGTI298 (results not shown). Therefore, inhibition of RAP1 activity appears to abrogate the actions of HC.

An interplay between the EPAC-RAP1 and HC signaling pathways was also identified by real-time PCR analyses (Figure 5B). EPAC and RAP1 mRNA levels were, respectively, 3.7-fold and 2.5-fold higher in HBMEC/ciβ cultured with CSC-HC than in CSC-free cultures. In addition, enhanced EPAC and RAP1 mRNA levels were not observed in CSC-HC cells when RU486 was present. These results suggest that the EPAC-RAP1 pathway plays a critical role in HC-mediated facilitation of AJ formation in HBMEC/ciβ.

### The differentiation status of HBMEC/ciβ was enhanced by hydrocortisone

**Figure 6:**
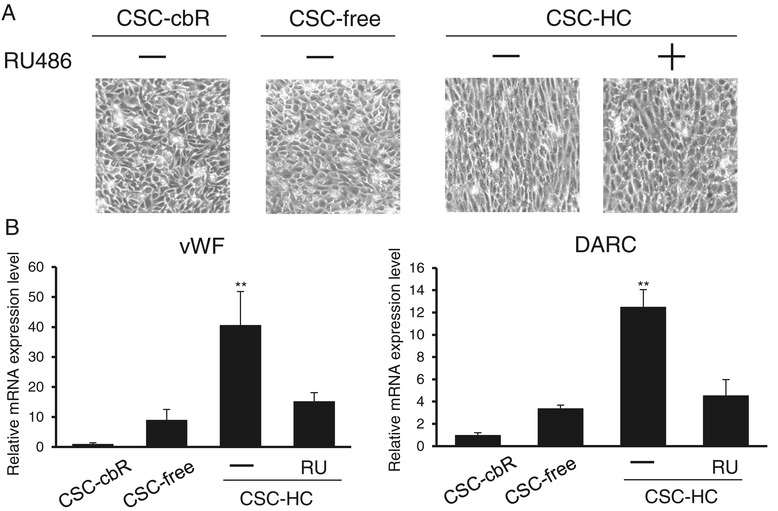
**Promotion of endothelial cell differentiation of HBMEC/ciβ by hydrocortisone. (A)** Cell morphology of HBMEC/ciβ cultured with CSC-cbR, CSC-free, or CSC-HC was analyzed by microscopy. The effect of RU486 on cell morphology was also tested using cells cultured with CSC-HC. The experiments were repeated four times and representative results are shown. **(B)** mRNA expression levels of representative endothelial cell differentiation marker genes (vWF and DARC) were determined by qPCR and calculated relative to the value obtained from CSC-cbR cells (set as basal level = 1). The “+” or “-” symbols indicate addition of RU486 or DMSO (0.1%) to the medium, respectively. Each value is expressed as mean ± S.D. obtained from three independent experiments. The single and double asterisks indicate *p* < 0.05 and *p* < 0.01, respectively, compared to CSC-free.

Proper AJ formation is generally associated with endothelial cell differentiation [15,22]. When cell morphology was examined during the above experiments, it was found that cells cultured with CSC-HC showed more spindle-like shapes with overall streamline contours, compared with cells cultured with other media (Figure 6A). However, this was not observed in the presence of RU486. Subsequently, the mRNA expression levels of endothelial differentiation marker genes vWF and DARC were analyzed (Figure 6B). The mRNA levels for both genes were significantly higher in CSC-HC cells than in those with other media. Thus, it appears that the endothelial cell differentiation of HBMEC/ciβ was promoted by HC through a GR pathway.

### HBMEC/ciβ migration ability was retarded by hydrocortisone

**Figure 7:**
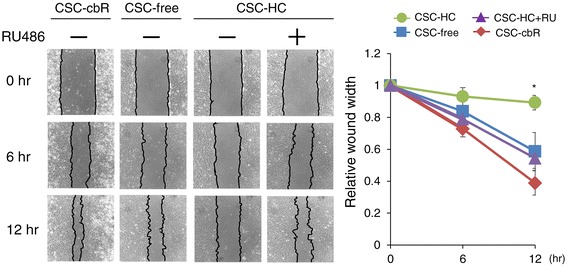
**Retardation of HBMEC/ciβ migration by hydrocortisone.** Migration abilities of HBMEC/ciβ cultured with CSC-cbR, CSC-free, or CSC-HC were examined by scratch assay. The effect of RU486 on cell morphology was also tested using cells cultured with CSC-HC. Wound width was determined under microscopic observation at 0, 6 and 12 hours after the scratch had been made. The experiments were repeated three times and the representative results are shown. In the right side, the relative value of wound width was calculated as the value obtained at time 0 (immediately after scratch) was set to 1, and each value is expressed as mean ± S.D. obtained from three independent experiments. The asterisk indicates *p* < 0.05 compared to the value of CSC-free.

It is known that endothelial cells reduce their growth activity and motility upon establishment of intercellular junctions [15]. Therefore, the migration ability of HBMEC/ciβ was examined by the scratch assay (Figure 7). The results showed that, as expected, the migration ability of the cells was retarded in the absence of cbR. Furthermore, it was found that addition of HC to the medium resulted in a further significant reduction in cell migration. The effects of HC were completely negated by RU486 treatment. Therefore, these results showed that cell migration was inhibited by HC via the GR function.

**Figure 8:**
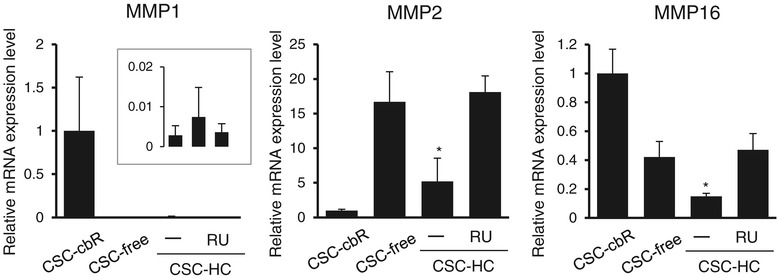
**Matrix metalloproteinase mRNA expression profile in HBMEC/ciβ cultured with each medium.** MMP-1, -2, and -16 mRNA expression levels in cells cultured with CSC-cbR, CSC-free, or CSC-HC were examined by qPCR. The effect of a GR antagonist, RU486, on HC-mediated modulation of mRNA expression was also investigated. The inset of the figure of MMP-1 shows magnified results obtained from cells cultured with CSC-free and CSC-HC (in the absence or presence of RU486) (left, middle, and right bar, respectively). mRNA expression levels were calculated relative to the value obtained from cells of CSC-cbR (set as basal level =1). Each value in the above experiments is expressed as mean ± S.D. obtained from three independent experiments. The “-” symbol indicates that DMSO (0.1%) was added to the medium as a control. The asterisk indicates *p* < 0.05 compared to the value of CSC-free.

A key contributor to cell motility is the family of MMPs that is capable of degrading the extracellular matrix in order to facilitate cell migration [23,24]. Thus, we hypothesized that the differential cell migration shown in Figure 7 would be associated with altered MMP expression. Since our preliminary microarray analysis had shown that, among the family members, MMP-1, MMP-2, and MMP-16 expressions were detected in HBMEC/ciβ (data not shown), the mRNA expression levels of these MMPs were examined in cells cultured with each medium (Figure 8). The results showed that cbR withdrawal from the medium caused more than 350-fold and 2-fold decrease in MMP-1 and MMP-16 mRNA levels, respectively, while it also led to more than 16-fold increase in MMP-2 mRNA levels. This might, at least partially, explain less significant reduction of migration ability observed in cells cultured with CSC-free conditions compared with those cultured in CSC-cbR. In addition, the results showed that HC treatment significantly reversed MMP-2 mRNA levels and further reduced MMP-16 mRNA levels (by 3.2-fold and 2.8-fold, respectively), while maintaining the repressive status of MMP-1 expression (Figure 8). Again, the effects of HC were prevented by RU486 treatment. These results were apparently consistent with the minimum migration ability of HBMEC/ciβ cultured with CSC-HC among the conditions, suggesting that altered MMP expression levels were involved in the effects of HC on HBMEC/ciβ mortality.

### Ether-type phosphatidylethanolamine levels were affected by hydrocortisone

**Figure 9:**
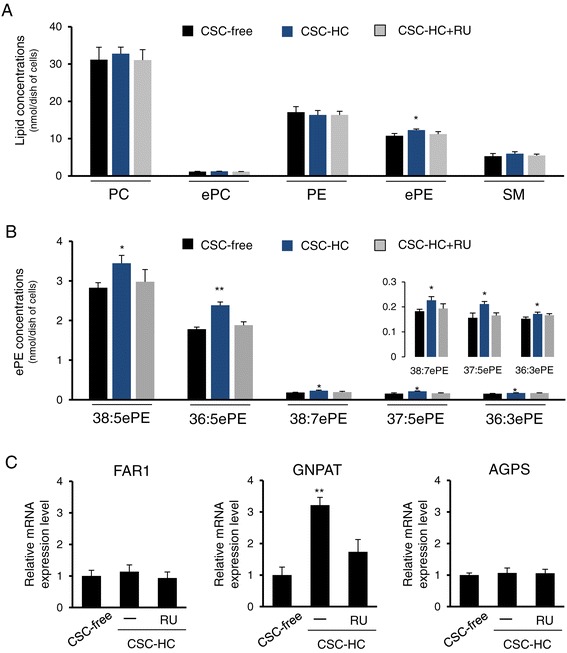
**Identification of unique ether-phosphatidylethanolamine increases in HBMEC/ciβ cultured with CSC-HC. (A)** Total lipids were extracted from cells cultured with CSC-free, CSC-HC or CSC-HC + RU486, and then subjected to LC-TOFMS analysis with a primary focus on the major lipid molecules that make up cell membranes. Each lipid concentration was normalized with that of the internal standard, and each bar represents a mean ± S.D. obtained from three independent experiments. **(B)** ePE concentrations were further determined using the same method. Among the twenty-one ePE molecules showing effective signal intensity, the results of five molecules (38:5ePE, 36:5ePE, 38:7ePE, 37:5ePE, and 36:3ePE) are shown. The inset shows magnified results of three minor species. Each lipid concentration was normalized with that of internal standard, and each bar represents a mean ± S.D. obtained from three independent experiments. **(C)** FAR1, GNPAT and AGPS mRNA expression levels in cells cultured with CSC-free or CSC-HC were examined by qPCR. The effect of RU486 on the HC-mediated induction of mRNA expression was also investigated. mRNA expression levels were calculated relative to the values obtained from CSC-free cells (set as the basal level =1). Values are expressed as means ± S.D. obtained from three independent experiments. In all the above, single and double asterisks indicate *p* < 0.05 and *p* < 0.01, compared to the CSC-free value.

Since the morphology and mobility of HBMEC/ciβ differed significantly depending on the presence and absence of HC, it was speculated that HC may actively influence the membrane lipid composition necessary to adapt to global cellular phenotypic alterations. Thus, a lipidomics approach, primarily focusing on major membrane lipid molecules (glycerophospholipids and sphingomyelin), was utilized in order to determine whether lipid composition differed among cells cultured with CSC-free, CSC-HC, or CSC-HC with RU486. While PC, ePC, PE, and SM levels were not significantly different among the conditions, ePE level in cells with CSC-HC was increased to a slight, but significant, degree compared to CSC-free (Figure 9A). Furthermore, this increase was mostly reversed by RU486 treatment.

Based on those results, detailed ePE molecule determination was conducted in order to identify the ePE species primarily affected by HC. The results showed that, among twenty-one molecules showing effective signal intensities, five molecules (38:5ePE, 36:5ePE, 38:7ePE, 37:5ePE, and 36:3ePE) were significantly increased in cells cultured with CSC-HC compared to CSC-free (Figure 9B). Furthermore, 38:5ePE and 36:5ePE were found to be the primary ePE species among the five (Figure 9B). It has previously been known that these lipid molecules have the 1-O-(1Z-alkenyl)-2-acyl-sn-glycerophosphatidylethanolamine structure. When the two acyl moieties of these molecules were searched for by referring to the reference data, 38:5ePE and 36:5ePE were found to be primarily derived from 18:0p/20:4 PE and 16:0p/20:4 PE, respectively. Next, the effects of HC on mRNA levels of three key ePE biosynthesis enzymes were examined. While FAR1 and AGPS mRNAs were not affected by HC, GNPAT mRNA levels in cells cultured with CSC-HC were significantly higher (over 3-fold) than cells cultured with CSC-free (Figure 9C). This enhancement was not detected in cells cultured with CSC-HC + RU486. Taken together, these results showed that HC significantly modulated cellular ePE levels, specifically 38:5ePE and 36:5ePE, partially via enhancing GNPAT mRNA levels.

## Discussion

Our results clearly show that culture media composition indeed plays an important role in determining HBMEC/ciβ functionality. Among the factors examined in this study, the supplementation of HC to a medium at a physiological concentration provides the highest beneficial impact on the barrier functionality of the HBMEC/ciβ-based *in vitro* BBB model. In addition to HC, our results showed that both the CSC basal medium and cbR likely affect HBMEC/ciβ biology. However, since details of cbR and CSC basal media composition have not been published and since neither was capable of inducing a notable functional improvement to the barrier property of HBMEC/ciβ, the remainder of this discussion will focus solely on the effects of HC.

HC-mediated enhancement of the HBMEC/ciβ barrier function is consistent with previous results obtained from porcine, mouse, rat, and human BMECs [25-28]. The Na-F P_app_ of cells cultured with CSC-HC (0.71 [×10^−3^ cm/min]) is comparable to that reported in rat or bovine primary cell-based BBB models (0.92 and 0.66 [×10^−3^ cm/min]) [29,30]. The Na-F P_app_ is also close to those obtained from other human-derived immortalized cell lines (hBMEC, hCMEC/D3, and TY08), which are 0.30 to 0.75 (×10^−3^ cm/min) [31]. Therefore, even though the different experimental conditions employed among those studies should be considered when interpreting their data, it is believed that improvement of the HBMEC/ciβ barrier function by HC significantly enhances their usability in various BBB studies.

It has been acknowledged that AJs play an important role in the control of vascular permeability [15,22], and it is thus considered likely that the barrier tightening effect of HC on HBMEC/ciβ is primarily mediated by facilitating AJ formation. On the other hand, our results also indicate immature TJ formation in the same culture condition. Because both AJs and TJs are essential for ensuring mature barrier integrity between cells through their reciprocal interaction, it seems that a critical point required for the achievement of further strengthening barrier tightness of HBMEC/ciβ lies in facilitating TJ formation. Since our present results are in accord with the notion that AJ formation precedes TJ assembly [22,32], the CSC-HC culture is presumed to provide HBMEC/ciβ with a cellular foundation for TJ formation. This indicates that additional stimuli that can be used to build up firm TJs in the cells should be sought in future experiments. These, if identified, can be expected to not only further enhance HBMEC/ciβ functionality, but also provide key clues towards understanding the molecular process of TJ formation. It has been shown that several soluble factors, such as retinoic acid, cAMP, and hepatocyte growth factor, along with co-culture with astrocytes and/or pericytes are capable of enhancing junctional property of *in vitro* BBB models in an independent or collaborative manner [28,33-35]. Therefore, it would be worthwhile to test these culture modifications in order to identify ways whereby TJ formation could be promoted. In such experiments, it may be important to consider crosstalk among media components in the optimization of HBMEC/ciβ culture method, because our results show that cbR apparently counteracts HC’s effects, even though the mechanism behind this observation is currently unclear.

Enhanced barrier tightness, AJ formation, spindle-like cell morphology, and abundant endothelial marker gene expression, are all signs of endothelial differentiation. In particular, AJ formation is critically important in not only stabilizing intercellular adhesion but also in promoting endothelial differentiation [15,22]. On the other hand, our results also show that cell migration is significantly disturbed by HC when it is associated with a reduction of MMP-2 and MMP-16 mRNA levels. In line with the reversed characteristics observed in HBMEC/ciβ cultured without HC (CSC-cbR or CSC-free), we suggest taking a comprehensive view of the phenotype transition; specifically, the mesenchymal-to-endothelial transition (MEndT), into consideration in order to gain a global picture of HC effects on HBMEC/ciβ. Recently, it has become evident that the plasticity of endothelial cells allows them to change their phenotype from endothelial to mesenchymal, and *vice versa*, which are regarded as the endothelial-to-mesenchymal transition (EndMT) and the MEndT, respectively [36]. EndMT molecular events are apparently similar to those of epithelial-to-mesenchymal transition (EMT) in epithelial cells, which includes abnormal intercellular junction formation, extensive cell migration, and remarkable expression of matrix remodeling genes [37]. Similarly, the reverse processes, the MEndT and the mesenchymal-to-epithelial transition (MET), appear to share numerous hallmarks, such as cell-to-cell junction stabilization, actin filament reorganization to the peri-plasma membrane region, cell migration ability retardation, and an increase in marker gene expression [37]. From this viewpoint, the overall effects of HC on HBMEC/ciβ appear to be closely related to MEndT. This is also consistent with previous results showing that glucocorticoid induces the MET or prevents the EMT in several cell types [38-40]. However, alterations in mRNA expression of some representative mesenchymal genes (such as α-smooth muscle actin) were not associated with phenotype transition in our cells (data not shown). Taken together, it appears that the effects of HC on HBMEC/ciβ can be regarded as “MEndT-like”. When this view is applied to the interpretations of previous literature, it appears that MEndT-like glucocorticoid effects have also been found in rat and mouse BMEC lines [41-43].

Because the MEndT/MET is a drastic phenotypic transition, it is not surprising that re-organization of various cellular processes are involved during the MEndT-like transition of HBMEC/ciβ. The present study, for the first time, verified that increased ePE levels are one of those processes. Although there have been no reports associating altered ePE levels with the MEndT/EndMT process, it has been reported that primary bovine aortic endothelial cells at the confluent state contain higher ePE levels than at the sub-confluent state [44], and that increased ePE levels were observed during the MET in MDCK cells [45]. Thus, our results may share similar findings with those reports. The ePE species identified here, 18:0p/20:4 PE and 16:0p/20:4 PE are classified as plasmalogens, and recent reports have shown that plasmalogens have diverse biological activities [46]. Among them, effects reducing membrane fluidity might be, at least partially, involved in lowering Na-F permeability and/or retarding mobility in HBMEC/ciβ cultured in CSC-HC. Nevertheless, it remains mostly unclear whether increased plasmalogen levels are adapted responses to (and/or necessary for) the HC-mediated MEndT-like process of HBMEC/ciβ. Therefore, progress in this new area of study has the potential to allow us to advance towards a greater understanding of a previously unacknowledged aspect of BBB biology and/or its barrier property.

Finally, the mechanisms by which an HC-GR-pathway facilitates AJ formation should be discussed. Based on our results, it appears that the EPAC-RAP1 pathway is a pivotal player involved in HC-mediated AJ protein translocation to the action site. Although it has been shown that the pathway is critically involved in AJ formation [21], our results are (to our knowledge) the first showing that the EPAC-RAP1 pathway can be located downstream of the HC-GR signaling pathway.

Considering that GR is a transcription factor, it is reasonable to assume that GR activation enhances EPAC and RAP1 mRNA levels in order to expand their potential activity upon stimulation, but it is difficult to believe that the GR function directly activates the EPAC-RAP1 activity. Rather, activated GR is likely to enhance or repress target gene expression, which in turn elicits various signals necessary to increase intracellular cAMP levels leading to EPAC-RAP1 pathway activation. Although it remains unknown what signaling pathways are activated by HC in order to stimulate an increase in cellular cAMP levels, we have observed that the expression levels of various transcription and autocrine/paracrine factors were targets of the HC-GR axis, as can be seen in the representative results (see Additional file 7: Figure S5). Therefore, it can be speculated that at least one of the targets might be involved in the intermediate signaling pathway(s) that bridge the gap between the HC-GR and EPAC-RAP1 pathways. Identifying the target(s) involved and the related signaling pathway(s) will be an interesting and important challenge in future studies, not only of HBMEC/ciβ but also of other BBB cells/cell lines.

## Conclusions

Our results show that HC clearly improves HBMEC/ciβ barrier functionality through GR activation, thus leading to our recommendation that HC be regarded as an essential media component for HBMEC/ciβ-based *in vitro* BBB models. It is also considered likely that these HC effects result from the orchestration of diverse cellular signaling networks, including those involved in modulating plasmalogen levels and accelerating AJ formation, which appears to be adapted to (and/or lead to) MEndT-like phenotype transitions. Nevertheless, since HBMEC/ciβ differentiation status, including TJ formation, is likely to be further promoted by optimization of culture conditions, we hope that such research efforts will result in the development of unique and useful HBMEC/ciβ-based *in vitro* BBB models, while simultaneously providing new opportunities to explore molecular events related to MEndT/EndMT in BMEC.

## Additional files

Additional file 1: Figure S1. Illustration of experimental procedure and medium information. Culture schedules are shown at the top. At Day 3, CSC-cbR was changed to fresh CSC-cbR, CSC-HC, or CSC-free medium, as indicated at the center of the illustration. All functional analyses, including immunocytochemistry, gene expression analyses, and Na-F permeability assays, were performed at Day 12. Medium composition is shown at the bottom. Additional file 2: Table S1. Primers used for qPCR in this study. Additional file 3: Table S2. Summary of identification of ether-phosphatidylethanolamine molecules (PC, ePC, PE, ePE and SM) in HBMEC/ciβ cells. Additional file 4: Figure S2. Exploration of cooperative effects of other SQs component(s) with hydrocortisone on barrier property of HBMEC/ciβ. CSC-HC supplemented with ascorbate and heparin, which are components of SQs, was used as a basic medium. Different SQs growth factor combinations were tested to determine if they had the potential to further enhance HBMEC/ciβ barrier properties. The “-” and “+” symbols indicate absence and presence of respective growth factors. Three days after cell seeding, the medium was changed to one of the two above-mentioned media. The cells were continuously cultured for 12 days, after which Na-F permeability assay was performed. The P_app_ value obtained from the cells cultured with CSC-HC in the absence of any growth factors was set to the basal level (=1) in each assay. Each bar represents the mean ± S.D. of the relative Na-F permeability values, which were obtained from three independent experiments. Additional file 5: Figure S3. VE-cadherin expression profile in HBMEC/ciβ. **A,** VE-cadherin mRNA expression in HBMEC/ciβ cultured with CSC-cbR or CSC-HC were determined by real-time PCR. Each value represents mean ± S.D. of three independent assays, each performed in duplicate. The mean value obtained from HBMEC/ciβ cultured with CSC-cbR was set to 1. **B,** VE-cadherin protein expressions in HBMEC/ciβ cultured with CSC-cbR or CSC-HC was determined by Western blot. β-actin protein expression was used as a loading control. The representative results of three independent assays are shown. Additional file 6: Figure S4. Claudin-5 and occludin expression profiles in HBMEC/ciβ. **A,** claudin-5 and occludin mRNA expressions in HBMEC/ciβ cultured with CSC-cbR or CSC-HC were determined by real-time PCR. Each value represents mean ± S.D. of three independent assays, each performed in duplicate. The mean value obtained from HBMEC/ciβ cultured with CSC-cbR was set as 1. **B,** claudin-5 and occludin protein expression in HBMEC/ciβ cultured with CSC-cbR or CSC-HC was determined by Western blot. β-actin protein expression was used as a loading control. The representative results of three independent assays are shown. Additional file 7: Figure S5. ANGPT2, Id-1, Egr-1 and HDAC7 mRNA expression in HBMEC/ciβ cultured with CSC-cbR, CSC-free, or CSC-HC. ANGPT2, Id-1, Egr-1 and HDAC7 mRNA expression levels in cells cultured with CSC-cbR, CSC-free, or CSC-HC, were examined by qPCR. The effect of a GR antagonist, RU486, on HC-mediated modulation of mRNA expression was also investigated. mRNA expression levels were calculated relative to the value obtained from CSC-cbR cells (set as basal level =1). Each value in the above experiments is expressed as mean ± S.D. obtained from three independent experiments. The “-” symbol indicates that DMSO (0.1%) was added to the medium as a control. The single and double asterisks indicate *p* < 0.05 and *p* < 0.01, respectively, compared with the value of CSC-free. ANGPT2 is a secreted glycoprotein member of the angiopoietin family of growth factors. It has been shown to be involved in functional impairment of the BBB [47]. Id-1 is a basic helix-loop-helix transcription factor family member, while lacking a basic DNA binding domain. It has been shown that Id-1 plays a facilitative role in EMT [48]. EGR-1 is a C2H2-class zinc finger transcription factor and its knockdown has been shown to be associated with retardation of cell migration ability [49]. HDAC7 is a member of the HDACs that contribute to gene transcription control via modification of acetylation level of histones, along with other proteins. It has been reported that HDAC7 mRNA knockdown causes reduction of endothelial migration [50].

## Abbreviations

AJ: Adherens junction
BBB: Blood–brain barrier
BMEC: Brain microvascular endothelial cells
EndMT (EMT): Endothelial (epithelial)-to-mesenchymal transition
EPAC: Exchange proteins activated by cAMP
ePE: Ether-phosphatidylethanolamine
GR: Glucocorticoid receptor
HC: Hydrocortisone
MEndT (MET): Mesenchymal-to-endothelial (epithelial) transition
MMP: Matrix metalloproteinase
Na-F: Sodium fluorescein
RAP1: Ras-proximate-1 or Ras-related protein 1
TEER: Transendothelial electric resistance
TJ: Tight junction
VE: Vascular endothelial
ZO: Zonula occludens
