# Supplementary material for: Hydrocortisone enhances the barrier properties of HBMEC/ciβ, a brain microvascular endothelial cell line, through mesenchymal-to-endothelial transition-like effects
Source: Fluids Barriers CNS. 2015 Mar 5;12:7. doi: 10.1186/s12987-015-0003-0 (PMC4355132; doi:10.1186/s12987-015-0003-0)
Supplement: Additional file 1: Figure S1. — Illustration of experimental procedure and medium information. Culture schedules are shown at the top. At Day 3, CSC-cbR was changed to fresh CSC-cbR, CSC-HC, or CSC-free medium, as indicated at the center of the illustration. All functional analyses, including immunocytochemistry, gene expression analyses, and Na-F permeability assays, were performed at Day 12. Medium composition is shown at the bottom. [file 12987_2015_3_MOESM1_ESM.pdf]

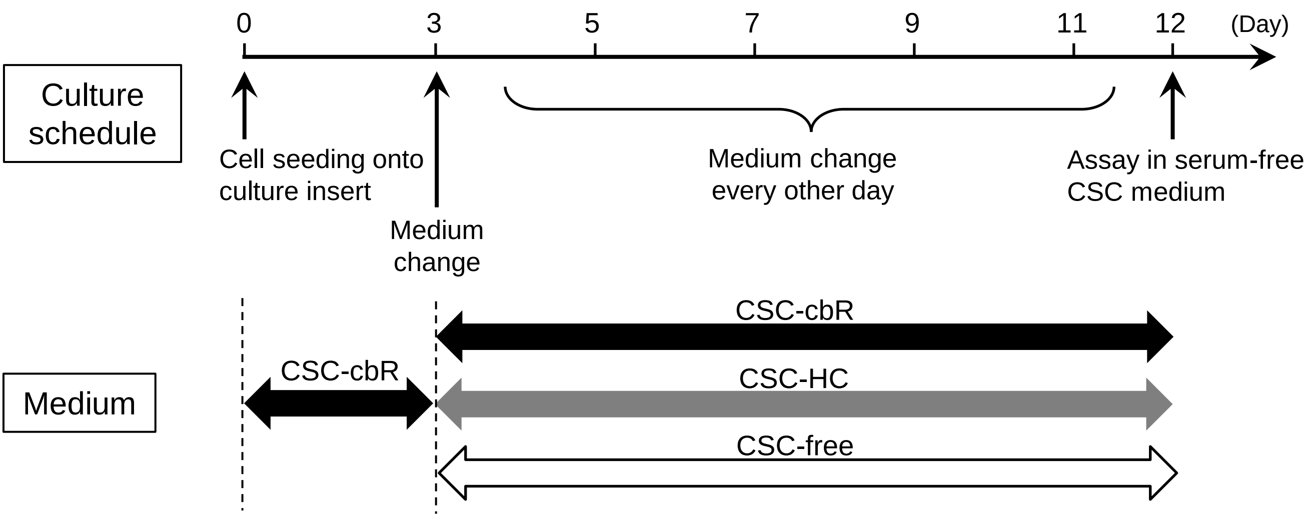

**Medium name**	**CSC-cbR**	**CSC-HC**	**CSC-free**
Basal medium	CSC medium with 10% serum	CSC medium with 10% serum	CSC medium with 10% serum
Culture supplements	CultureBoost-R	HC (180 nM)	None

Figure S1
