# Supplementary material for: Hydrocortisone enhances the barrier properties of HBMEC/ciβ, a brain microvascular endothelial cell line, through mesenchymal-to-endothelial transition-like effects
Source: Fluids Barriers CNS. 2015 Mar 5;12:7. doi: 10.1186/s12987-015-0003-0 (PMC4355132; doi:10.1186/s12987-015-0003-0)
Supplement: Additional file 2: Table S1. — Primers used for qPCR in this study. [file 12987_2015_3_MOESM2_ESM.pdf]

Table S1. Primers used in this study

Primer name[a]	Sequence (5’ > 3’)
ANXA1 F	TGCTAAGGGTGACCGATCTGAG
ANXA1 R	CTTTCTCCTGCTTCATACAAGGC
GLIZ F	CCATCCTGCTCTTCTTCCAC
GLIZ R	ATGGCCTGTTCGATCTTGTT
NFkBIA F	GTCAAGGAGCTGCAGGAGAT
NFkBIA R	ATGGCCAAGTGCAGGAAC
vWF F	AGT GCA GAC CCA ACT TCA CC
vWF R	GTG GGG ACA CTC TTT TGC AC
DARC F	TGATGGCCCTCATTAGTCCT
DARC R	TTCTCAGTTGAGGGGGAGAG
MMP1 F	GCTAACCTTTGATGCTATAACTACGA
MMP1 R	TTTGTGCGCATGTAGAATCTG
MMP2 F	AGAAGGCTGTGTTCTTTGCAG
MMP2 R	AGGCTGGTCAGTGGCTTG
MMP16 F	AGGGCATCCAGAAGATATATGG
MMP16 R	GGCACTGTCGGTAGAGGTCTT
ANGPT2 F	TGCAAATGTTCACAAATGCTAA
ANGPT2 R	AAGTTGGAAGGACCACATGC
Id-1 F	CCAGAACCGCAAGGTGAG
Id-1 R	GGTCCCTGATGTAGTCGATGA
EGR-1 F	AGCCCTACGAGCACCTGAC
EGR-1 R	GGTTTGGCTGGGGTAACTG
HDAC7 F	CTCCAGCAGCACCCTCAG
HDAC7 R	CCAGAGGAAGCAGCACAGT
EPAC1 F	TGTGCGAGCTTGAAAAGAAA
EPAC1 R	CTCCCAGAGCTGGGCTATC
RAP1A F	TGGAAAAGAAGAAGCCTAAAAAGA
RAP1A R	CAGTTCTTCATTCCTGTAATCTGG
GNPAT F	AAACCTTTTGTGGAAAGCTATCAG
GNPAT R	CTGACTGCAGCCAAGTACTGTT
AGPS F	CTTCAACATGAAAAACAAGTGTATGA
AGPS R	CAGCAAATAACCTCTCTGTCCA
FAR1 F	CTGCTTGGGAACTAAGAAGTACG
FAR1 R	CCATAACGTATATTCCGCAACTT

VE-cadherin F	AATGCGTCCGTGCCTGAGTCGT
VE-cadherin R	GTGGTCTCCCACAGTGGGGTCG
Occludin F	AGGAACCGAGAGCCAGGT
Occludin R	GGATGAGCAATGCCCTTTAG
Claudin-5 F	CTTCCAGAATGGCAAGAGAGTGA
Claudin-5 R	ACCACTGTTCTCCACTGCTCAGA
GAPDH F	AGCCACATCGCTCAGACAC
GAPDH R	GCCCAATACGACCAAATCC

[a], F and R indicate forward and reverse direction.
