# Supplementary figures and images for: Hydrocortisone enhances the barrier properties of HBMEC/ciβ, a brain microvascular endothelial cell line, through mesenchymal-to-endothelial transition-like effects
Source: Fluids Barriers CNS. 2015 Mar 5;12:7. doi: 10.1186/s12987-015-0003-0 (PMC4355132; doi:10.1186/s12987-015-0003-0)

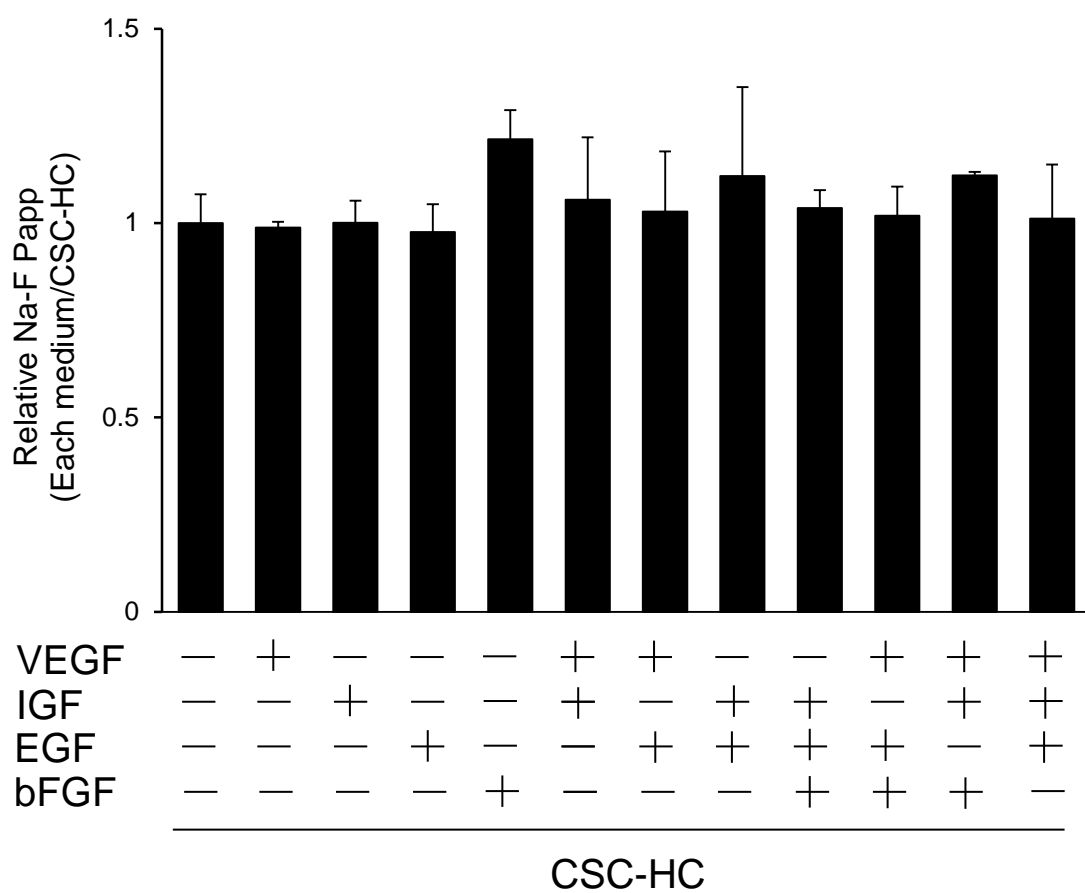

Figure S2

Supplement: Additional file 4: Figure S2. — Exploration of cooperative effects of other SQs component(s) with hydrocortisone on barrier property of HBMEC/ciβ. CSC-HC supplemented with ascorbate and heparin, which are components of SQs, was used as a basic medium. Different SQs growth factor combinations were tested to determine if they had the potential to further enhance HBMEC/ciβ barrier properties. The “-” and “+” symbols indicate absence and presence of respective growth factors. Three days after cell seeding, the medium was changed to one of the two above-mentioned media. The cells were continuously cultured for 12 days, after which Na-F permeability assay was performed. The Papp value obtained from the cells cultured with CSC-HC in the absence of any growth factors was set to the basal level (=1) in each assay. Each bar represents the mean ± S.D. of the relative Na-F permeability values, which were obtained from three independent experiments. [file 12987_2015_3_MOESM4_ESM.pdf]

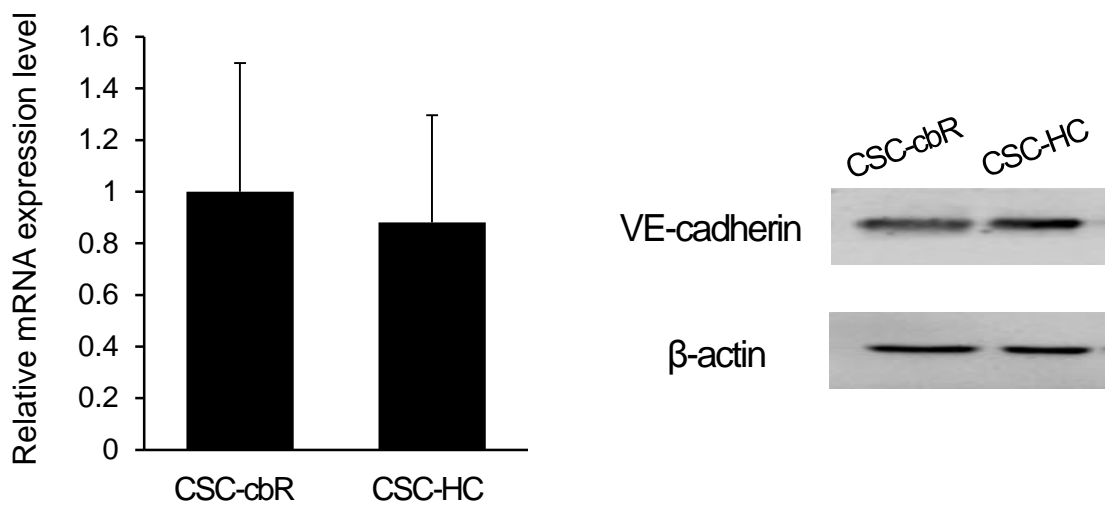

Figure S3

Supplement: Additional file 5: Figure S3. — VE-cadherin expression profile in HBMEC/ciβ. A, VE-cadherin mRNA expression in HBMEC/ciβ cultured with CSC-cbR or CSC-HC were determined by real-time PCR. Each value represents mean ± S.D. of three independent assays, each performed in duplicate. The mean value obtained from HBMEC/ciβ cultured with CSC-cbR was set to 1. B, VE-cadherin protein expressions in HBMEC/ciβ cultured with CSC-cbR or CSC-HC was determined by Western blot. β-actin protein expression was used as a loading control. The representative results of three independent assays are shown. [file 12987_2015_3_MOESM5_ESM.pdf]

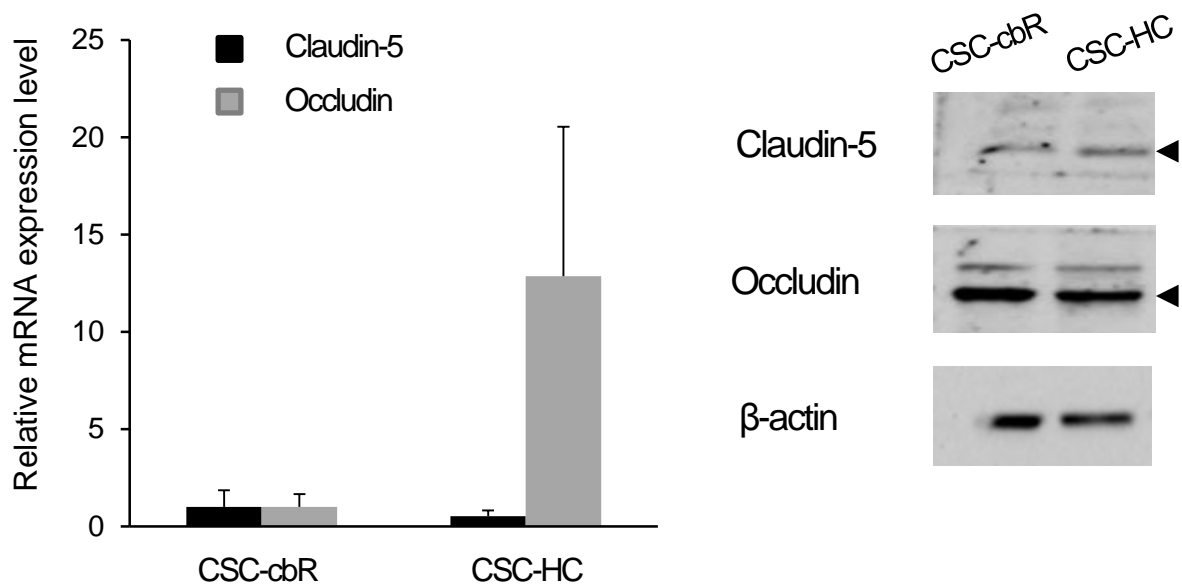

Figure S4

Supplement: Additional file 6: Figure S4. — Claudin-5 and occludin expression profiles in HBMEC/ciβ. A, claudin-5 and occludin mRNA expressions in HBMEC/ciβ cultured with CSC-cbR or CSC-HC were determined by real-time PCR. Each value represents mean ± S.D. of three independent assays, each performed in duplicate. The mean value obtained from HBMEC/ciβ cultured with CSC-cbR was set as 1. B, claudin-5 and occludin protein expression in HBMEC/ciβ cultured with CSC-cbR or CSC-HC was determined by Western blot. β-actin protein expression was used as a loading control. The representative results of three independent assays are shown. [file 12987_2015_3_MOESM6_ESM.pdf]

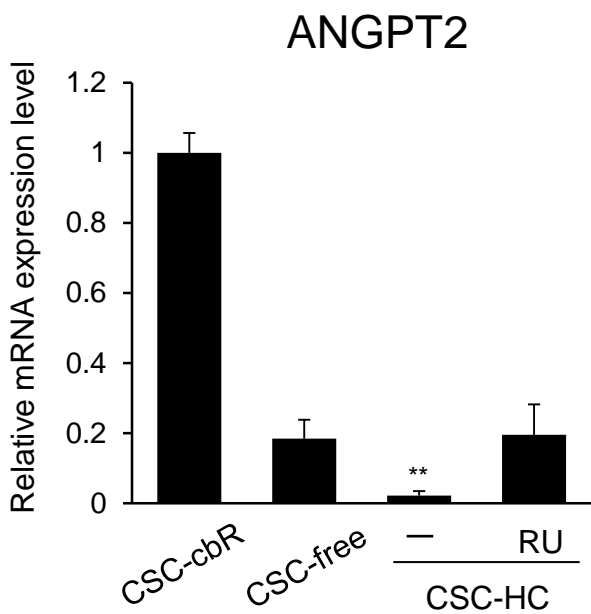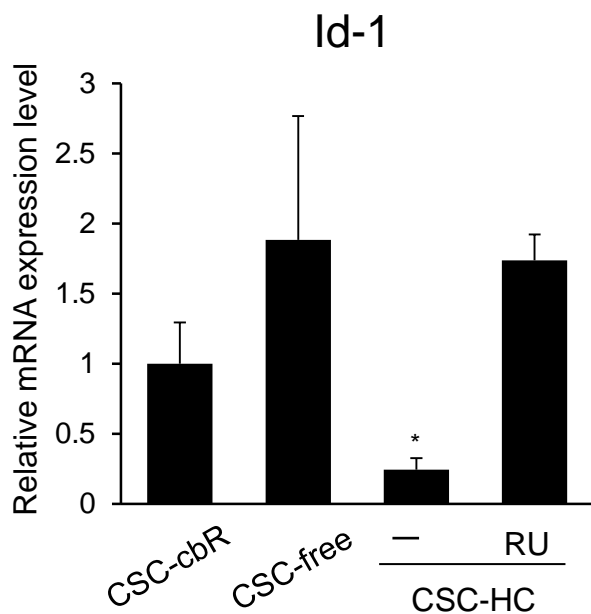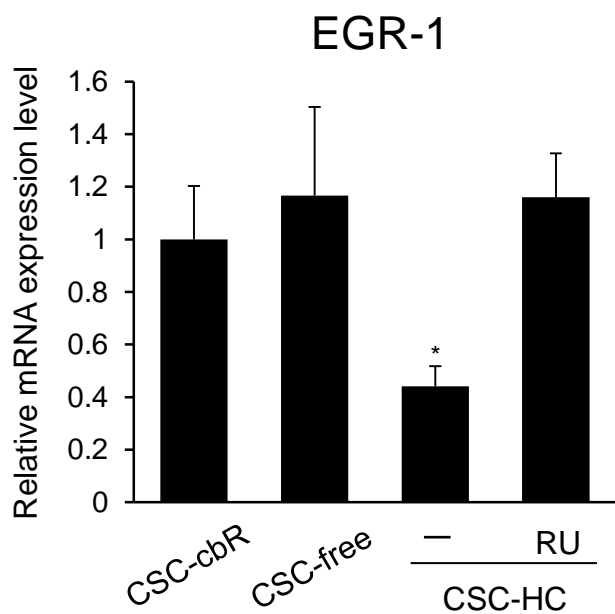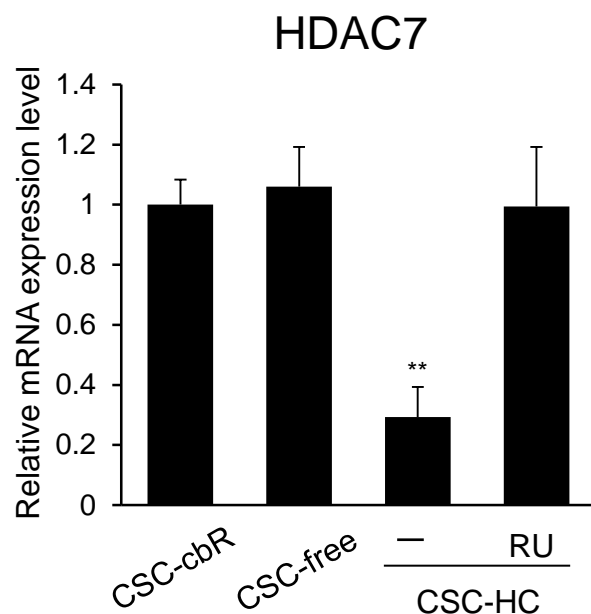

Figure S5

Supplement: Additional file 7: Figure S5. — ANGPT2, Id-1, Egr-1 and HDAC7 mRNA expression in HBMEC/ciβ cultured with CSC-cbR, CSC-free, or CSC-HC. ANGPT2, Id-1, Egr-1 and HDAC7 mRNA expression levels in cells cultured with CSC-cbR, CSC-free, or CSC-HC, were examined by qPCR. The effect of a GR antagonist, RU486, on HC-mediated modulation of mRNA expression was also investigated. mRNA expression levels were calculated relative to the value obtained from CSC-cbR cells (set as basal level =1). Each value in the above experiments is expressed as mean ± S.D. obtained from three independent experiments. The “-” symbol indicates that DMSO (0.1%) was added to the medium as a control. The single and double asterisks indicate p < 0.05 and p < 0.01, respectively, compared with the value of CSC-free. ANGPT2 is a secreted glycoprotein member of the angiopoietin family of growth factors. It has been shown to be involved in functional impairment of the BBB [47]. Id-1 is a basic helix-loop-helix transcription factor family member, while lacking a basic DNA binding domain. It has been shown that Id-1 plays a facilitative role in EMT [48]. EGR-1 is a C2H2-class zinc finger transcription factor and its knockdown has been shown to be associated with retardation of cell migration ability [49]. HDAC7 is a member of the HDACs that contribute to gene transcription control via modification of acetylation level of histones, along with other proteins. It has been reported that HDAC7 mRNA knockdown causes reduction of endothelial migration [50]. [file 12987_2015_3_MOESM7_ESM.pdf]
